# Intersecting Memories of Immunity and Climate: Potential Multiyear Impacts of the El Niño–Southern Oscillation on Infectious Disease Spread

**DOI:** 10.1029/2024GH001193

**Published:** 2025-02-11

**Authors:** Maya V. Chung, Gabriel A. Vecchi, Wenchang Yang, Bryan Grenfell, C. Jessica Metcalf

**Affiliations:** ^1^ Program in Atmospheric and Oceanic Sciences Princeton University Princeton NJ USA; ^2^ High Meadows Environmental Institute Princeton University Princeton NJ USA; ^3^ Department of Geosciences Princeton University Princeton NJ USA; ^4^ Department of Ecology and Evolutionary Biology Princeton University Princeton NJ USA; ^5^ Princeton School of Public and International Affairs Princeton NJ USA

**Keywords:** infectious disease, ENSO, climate variability, disease forecasting

## Abstract

Climate and infectious diseases each present critical challenges on a warming planet, as does the influence of climate on disease. Both are governed by nonlinear feedbacks, which drive multi‐annual cycles in disease outbreaks and weather patterns. Although climate and weather can influence infectious disease transmission and have spawned rich literature, the interaction between the independent feedbacks of these two systems remains less explored. Here, we demonstrate the potential for long‐lasting impacts of El Niño–Southern Oscillation (ENSO) events on disease dynamics using two approaches: interannual perturbations of a generic SIRS model to represent ENSO forcing, and detailed analysis of realistic specific humidity data in an SIRS model with endemic coronavirus (HCoV‐HKU1) parameters. Our findings reveal the importance of considering nonlinear feedbacks in susceptible population dynamics for predicting and managing disease risks associated with ENSO‐related weather variations.

## Key Points

- El Niño‐Southern Oscillation (ENSO) impacts on infectious disease outbreaks may last multiple years via lagged nonlinear effects on population immunity
- Lagged immune response may cause larger changes in disease the year after an ENSO event or after consecutive ENSO events
- Population immunity should therefore be considered when investigating the impacts of ENSO on infectious disease

## 1 Introduction

Climate and weather can drive infectious disease dynamics through several mechanisms. Temperature, precipitation, and humidity changes can influence the spread of vector‐borne, water‐borne, and airborne diseases (Thomson, Grace, et al., 2018). Extreme weather events such as droughts, floods, and hurricanes can impact disease transmission by affecting water quality, disrupting healthcare systems, or triggering human migration (Mahmud et al., 2020).

The El Niño‐Southern Oscillation (ENSO) phenomenon is a well‐known driver of infectious disease‐relevant weather variables across the globe. ENSO oscillates between warm phases (El Niño) and cold phases (La Niña) approximately every 2–7 years (McPhaden et al., 2020). Its teleconnections with temperature, precipitation, and drought have been hypothesized to affect the spread of several vector and waterborne diseases, including cholera, dengue, malaria, hantavirus, plague, and Rift Valley fever (Anyamba et al., 2019). For example, during the strong 2015–2016 El Niño event, there were notable increases in disease activity in the Americas, southern Asia, and eastern Africa, especially in the tropics (Anyamba et al., 2019).

The long‐lead prediction capability of ENSO offers significant opportunities for disease forecasting. Unlike local weather predictions, which typically span days to a week, ENSO events can often be forecasted several months in advance (NOAA, 2024b). This advanced knowledge could facilitate seasonal health policy interventions such as vaccination campaigns and vector control efforts (Anyamba et al., 2022; Thomson, Metcalf, & Mason, 2018). The potentially widespread geographic impacts of ENSO‐related disease outbreaks also provide opportunities for health resource management and international collaborations to mitigate disease risks (Anyamba et al., 2019).

Despite this opportunity, few successful public health interventions have been based on ENSO‐disease forecasts. One exception was early warnings of high Rift Valley fever (RVF) risk associated with the 2006–2007 El Niño that led to increased vector and disease surveillance for 2–6 weeks before the first cases of the outbreaks in Kenya and Tanzania (Anyamba et al., 2009, 2010). Even longer‐lead early warnings allowed for preemptive livestock vaccination campaigns to prepare for the impacts of the 2015–2016 El Niño, which likely prevented the RVF outbreak that year (Anyamba et al., 2019).

The rarity of successful disease‐forecasting applications may reflect an important barrier around ENSO‐disease interactions: the complex relationships potentially involved. Factors such as different disease sensitivities to climate, geographical variations in ENSO's impact on weather, and the lag between ENSO events and disease seasons can add complexity to ENSO‐disease relationships (McGregor & Ebi, 2018). There is also evidence that the relationship between ENSO and disease may be non‐stationary, potentially due to ENSO's multi‐decadal variability or climate change; this was found to coincide with long‐term variations in cholera outbreaks in Bangladesh (Rodo et al., 2002).

Furthermore, several other factors can obscure the influence of ENSO on disease dynamics, such as local weather variations, human behavior, healthcare interventions, vector ecology, changes in disease surveillance, and background population immunity (e.g., Koelle et al., 2005; Martinez et al., 2017; McGregor & Ebi, 2018; Metcalf et al., 2017; Pascual et al., 2000; Tian et al., 2022). Consequently, regional studies aiming to establish statistical relationships between ENSO and disease have often produced mixed results (McGregor & Ebi, 2018). To better understand the footprint on ENSO on disease transmission, mechanistic modeling approaches are likely necessary.

Several mechanistic modeling studies have shown that population immunity can modulate the impact of ENSO and other environmental drivers on disease dynamics. Although weather and climate variability have been found to be important extrinsic drivers of disease, intrinsic disease dynamics are also significant, often with both adding skill to disease predictions but differing in their dominance across contexts (e.g., Koelle et al., 2005; Laneri et al., 2010; Pascual et al., 2008). Limitations in measurement of core quantities associated with infectious diseases (e.g., underestimation of infection incidence as a result of asymptomatic infection, or a widespread lack of measurement of immune status (Mina et al., 2020)) considerably enhance the value of mechanistic models to provide critical insights into infectious disease dynamics (Ionides et al., 2006; King et al., 2008; Laneri et al., 2010).

Despite these advances, the cumulative effects of experiencing consecutive climate anomalies and the temporal extent of these impacts remain underexplored. Given ENSO's multi‐year climate memory and the long‐term memory of immunity, the influence of ENSO on disease dynamics may be more predictable, complex, and long‐lasting than previously recognized. Furthermore, more research is needed on the interactions between ENSO and respiratory pathogens, which have received less attention compared to waterborne and vector‐borne diseases. Studies have reported associations between ENSO and seasonal influenza (e.g., Flahault et al., 2004; Oluwole, 2015; Viboud et al., 2004), influenza pandemics (Shaman & Lipsitch, 2013), and viral pneumonia hospitalizations (Ebi et al., 2001). However, many of these studies have been correlative and would benefit from further mechanistic exploration.

In this study, we build on previous work (e.g., Koelle et al., 2005; Pascual et al., 2000, 2008) to explore potential relationships between ENSO and infectious disease outbreaks using generalized mechanistic modeling approaches based on respiratory disease transmission. To move beyond the limitations of previous local‐scale, disease‐specific studies, we develop a susceptible‐infected‐recovered‐susceptible (SIRS) framework that explicitly captures cycles of infection and recovery and can be adapted to simulate various climate‐dependent diseases. Our methodology involves two modeling approaches. First, we use an SIRS model with a seasonally varying basic reproduction number $\left(R_{0}\right)$ that is interannually perturbed to investigate how the direct effects of ENSO on $R_{0}$ and more indirect effects on the susceptible population impact disease spread, and how these vary for different sequences of ENSO events. Second, we couple an SIRS model for an airborne disease, the endemic coronavirus HCoV‐HKU1, with specific humidity reanalysis data from 1981 to 2017 to examine the influence of ENSO‐related humidity variations on disease transmission. This approach follows evidence of the specific humidity dependence of influenza and respiratory syncytial virus (RSV) (Baker et al., 2019; Lowen & Steel, 2014; Lowen et al., 2007; Shaman & Kohn, 2009; Shaman et al., 2010).

The manuscript proceeds as follows: The methods section outlines our theoretical framework and modeling approaches. In Part 1 of the results section, we discuss the findings from the idealized SIRS‐ENSO model for a seasonal disease, followed by Part 2 for a biennial disease. In Part 3 we present the results from an HCoV‐HKU1 SIRS model forced by estimates of historical humidity (the MERRA‐2/SIRS model), including global maps of changes in infections associated with ENSO and a detailed analysis of the large impacts in northern Australia. Finally, similarities between the results of both modeling approaches are discussed, particularly emphasizing the multi‐year effects of ENSO events on infections driven mainly by changes in the susceptible population.

## 2 Methods

### 2.1 SIRS Model and ENSO‐Disease Theoretical Framework

The susceptible‐infected‐recovered‐susceptible (SIRS) model underpinning our ENSO‐disease models is defined by the following equations:

$$\frac{dS}{dt}=\xi R-\beta SI$$

$$\frac{dI}{dt}=\beta SI-\gamma I$$

S+I+R=1

where d*S*/d*t* is the time tendency of the susceptible fraction of the population, d*I*/d*t* is the time tendency of the infected fraction, R is the recovered fraction, ξ is the rate of immunity loss, β is the transmission rate, and γ is the recovery rate (Keeling & Rohani, 2008). The basic reproduction number is given by $R_{0}=\beta /\gamma$ and represents the contagiousness of a disease as the number of secondary infections expected to be caused by a single infection in a completely susceptible population (Dietz, 1993).

The analysis is grounded in human coronavirus SIRS models due to their contemporary relevance and known climate dependencies. These models neglect vital dynamics, as in Baker et al. (2020), but future work could examine the full family of SIR models, which include births and deaths.

**Figure 1:**
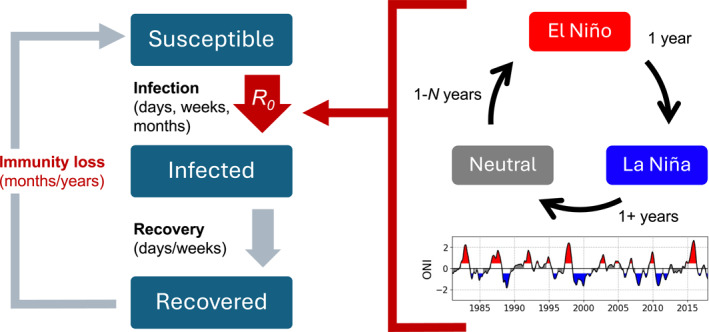
Schematic of how infectious disease and El Niño‐Southern Oscillation (ENSO) memory may interact. Left: An SIRS model with red parts indicating components that may interact with ENSO. Upper right: A typical ENSO progression between the warm phase (El Niño), cold phase (La Niña), and neutral state. ENSO does not always behave according to this sequence, but this sequence often occurs. Lower right: Oceanic Niño Index (ONI) from 1981 to 2017, with red shading indicating El Niño events, blue indicating La Niña events, and gray indicating the neutral state (NOAA, 2024a).

Figure 1 illustrates potential interactions between ENSO and infectious disease transmission through two mechanisms. First, ENSO can induce changes in $R_{0}$ within a season or year via its teleconnections to weather. These changes in $R_{0}$ could occur through several pathways related to temperature, precipitation, and humidity. Second, if the immunity loss timescale of a disease is sufficiently long, the interannual variability of ENSO can overlap with this timescale and indirectly affect multiple disease seasons by altering the susceptible population. Successive ENSO events may have compounding impacts on disease outbreaks through this mechanism, with potentially more complex impacts for biennial diseases than seasonal diseases.

Note that the ENSO cycle depicted in Figure 1 does not fully capture the irregular behavior of ENSO, as evidenced by the Oceanic Niño Index (ONI) timeseries also presented. While it has been observed that La Niña events are often preceded by strong El Niño events (Iwakiri & Watanabe, 2021), this pattern is not consistently observed. It is not uncommon to skip the neutral state or have multi‐year El Niño or La Niña events. The purpose of this figure is to illustrate the potential multi‐year dynamics of ENSO and its potential overlap with the immunity loss timescale as the ENSO state fluctuates.

### 2.2 Idealized SIRS‐ENSO Model

To investigate potential impacts of ENSO on infectious disease outbreaks, SIRS models are perturbed with idealized interannual ENSO forcing. Separate SIRS models are constructed to represent seasonal and biennial disease dynamics. Seasonal disease dynamics are modeled by varying $R_{0}$ seasonally, while biennial dynamics incorporate a longer immunity length and larger seasonal variations in $R_{0}$. The disease parameters for each experiment are summarized in Table 1. These parameters were chosen without correspondence to a specific disease.

**Table 1:** Disease Parameters Used for the Seasonal and Biennial SIRS Models

	Seasonal model	Biennial model
Immunity length (1/ξ)	500 days	3 years
Infection period (1/γ)	10 days	
Baseline $R_{0}$	3	
Seasonal $R_{0}$ variation amplitude	0.75	1.5
ENSO kick to $R_{0}$	0.75	

The seasonal and biennial SIRS models are run with a daily time step. Before applying ENSO perturbations, the models are spun up for 1,000 years to reach a stable attractor, though the models appear to stabilize within 20 (seasonal model) or 50 (biennial model) years. The interannual ENSO event‐like perturbations to $R_{0}$ begin between 11 and 14 years after the 1,000‐year spin‐up period, depending on the number of ENSO events in the scenario.

To simulate the impact of El Niño and La Niña events, $R_{0}$ is respectively increased or decreased by 0.75 within the boundaries of an ENSO event year and then is instantly returned to previous levels. Though this approach leads to unrealistic discontinuities, its simplicity offers clarity as to the footprint of such effects. To test the effects of consecutive ENSO events, we simulate various sequences including a single El Niño or La Niña event, one El Niño preceded by one and three La Niña events, and one La Niña preceded by one and three El Niño events. Note that the sequence of three El Niño events directly preceding a La Niña has not occurred in the real world in the modern ENSO record, but it is included for completeness.

To investigate how the timing of ENSO perturbations relative to the seasonal cycle of $R_{0}$ affects disease dynamics, we conduct two sets of experiments where the idealized ENSO perturbations are applied at different times in the year. The standard version aligns the start time of ENSO events = when $R_{0}$ is near its average value and is increasing seasonally, and a 6‐month shifted set of experiments offset the start of the ENSO perturbations 6 months later, when $R_{0}$ is near its average value and decreasing seasonally.

**Figure 2:**
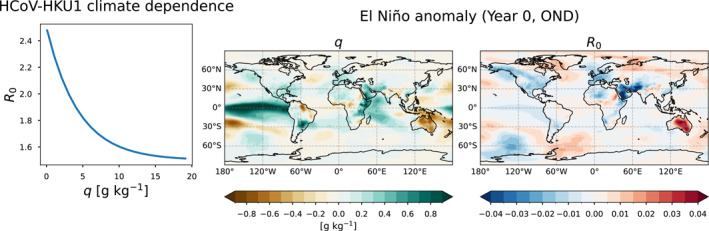
MERRA‐2/SIRS model methodology and El Niño composites. Left: Prescribed dependence of $R_{0}$ on specific humidity (q) based on previous studies. Center/Right: El Niño composites of specific humidity and $R_{0}$ shown as the October‐November‐December average anomaly from the monthly climatology.

It is important to note that potential impacts of ENSO on $R_{0}$ likely vary in sign, amplitude, and lag time across locations, and this idealized model simulates a subset of these possibilities. In the idealized model setup we chose to associate higher $R_{0}$ with El Niño and lower $R_{0}$ with La Niña because a strong signal with the same sign emerged in Australia in the MERRA‐2/SIRS model (Figure 2), where El Niño is associated with drier conditions and La Niña with wetter conditions. Reversing the sign of this relationship would yield the same results except “La Niña” and “El Niño” would be reversed. The standard‐timing version of the experiments is also similar to the timing of the ENSO‐related $R_{0}$ changes in Australia in the MERRA‐2/SIRS model, but they are not exactly the same because lag times of effects of ENSO on local weather can vary from event to event. The purpose of this simplified model is not to represent a particular location, but rather to explore how ENSO‐driven interannual variations in $R_{0}$ affect infections and susceptibles, to help interpret the behavior of a more complex system.

### 2.3 MERRA‐2/SIRS Model

To more realistically explore the relationship between ENSO and disease spread, an SIRS model is forced with historical weather data via specific humidity. We use the climate‐dependent transmission and immunity length parameters for HCoV‐HKU1, estimated in Baker et al. (2020) by fitting an SIRS model to HCoV‐HKU1 case data from the United States (CDC, 2020) while keeping all other parameters fixed based on values from Kissler et al. (2020). The relationship between specific humidity and $R_{0}$ based on these parameters is shown in Figure 2. The immunity length (1/ξ) is 66.25 weeks, and the infection period (1/γ) is 5 days.

Although climate drivers are likely to have broadly consistent effects on transmission for specific infections, differences in other drivers of transmission such as human behavior mean that applying disease model parameters derived from one country to the entire globe is unrealistic. However, our aim is not to provide an accurate forecast of disease incidence in a particular setting, but rather to explore potential interactions of population immunity with interannual climate forcing. Accordingly, to isolate potential effects of ENSO while avoiding complications related to local disease dynamics and human behavior, we apply the same disease model over all locations.

The SIRS model is forced with weekly mean 2 m specific humidity data with global coverage from the re‐analysis data set Modern‐Era Retrospective analysis for Research and Applications, Version 2 (MERRA‐2) produced by NASA's Global Modeling and Assimilation Office (Gelaro et al., 2017). The SIRS model is initialized with 1 infection per 100,000 people and the remaining fraction of the population as susceptible. The model is stepped forward using a 4th order Runge‐Kutta discretization. We “spin up” the disease model by forcing it with 20 years of repeated weekly MERRA‐2 climatology, which was averaged over the years 1980–2018. The infected and susceptible fractions at the end of the spin‐up are the initial conditions for the model run that is ultimately analyzed, which is forced with the MERRA‐2 specific humidity data from 1981 to 2017.

The model is not very sensitive to the length of the climatological spin‐up period, as it stabilizes after a few years for these parameters. We also investigated whether a 21‐year spin‐up period would affect regions with biennial disease, but there was very little difference, providing evidence that the 1981–2017 run is being strongly forced by the MERRA‐2 data.

ENSO event years between 1981 and 2017 are determined based on the Oceanic Niño Index (NOAA, 2024a). The ONI is a timeseries of 3 months running mean sea surface temperature anomalies in the Niño 3.4 region (5°N–5°S, 120°–170°W), where anomalies are computed relative to a 30‐year base period updated every 5 years. El Niño and La Niña events are determined when the ONI exceeds 0.5°C or falls below −0.5°C, respectively. Calendar years ending during El Niño events are classified as El Niño years (1982, 1986, 1987, 1991, 1994, 1997, 2002, 2004, 2006, 2009, 2014, 2015; 12 years total), while those ending during La Niña events are categorized as La Niña years (1983, 1984, 1988, 1995, 1998, 1999, 2000, 2005, 2007, 2008, 2010, 2011, 2016, 2017; 14 years). All other years are considered neutral years (11 years).

This classification follows the common practice for computing ENSO composites, where the first year (year 0) is counted as the calendar year during which the ENSO event ramps up in the Boreal Fall, and the event tends to extend into the next year (year +1) (Harrison & Larkin, 1998; Larkin & Harrison, 2005; Rasmusson & Carpenter, 1982). Some results also show the following year (year +2), which may coincide with the continuation of the original ENSO event or a different ENSO state.

## 3 Results

### 3.1 Seasonal SIRS Model

**Figure 3:**
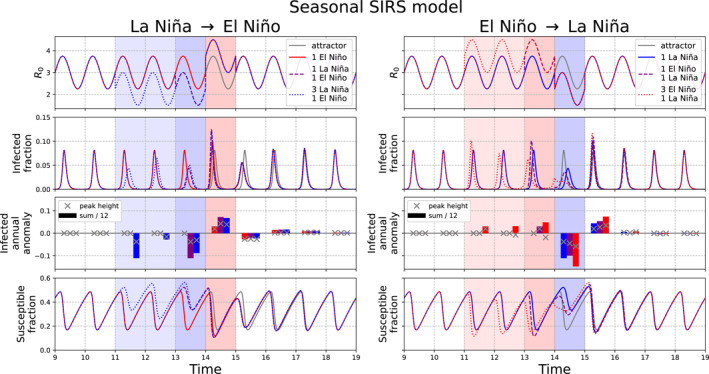
SIRS model response to El Niño‐Southern Oscillation (ENSO) perturbations for El Niño and preceding 1 or 3 La Niña events (left) and La Niña and preceding 1 or 3 El Niño events (right). All the models have seasonally varying $R_{0}$, with no interannual perturbations to the attractor (gray), and with simulated El Niño (pink shading) and La Niña events (blue shading) as a 1‐year $R_{0}$ increase or decrease from the seasonally varying baseline, respectively. Darker shading indicates the overlap in timing between the 1 and 3 preceding‐event scenarios. Quantities shown over time are $R_{0}$, infected fraction, infected fraction annual anomaly from the attractor as the difference in peak height (×) and annual sum divided by 12 (bars), and susceptible fraction. Colors for the infected annual anomalies correspond to the ENSO event scenarios with line plots of the same color. Left: One El Niño event (red solid line), La Niña followed by El Niño (purple dashed line), and three La Niña events followed by El Niño (blue dotted line). Right: One La Niña event (blue line), El Niño followed by La Niña (purple dashed line), and three El Niño events followed by La Niña (red dotted line). Time shown in years after 1,000‐year spin‐up.

Figure 3 illustrates how various sequences of idealized ENSO events affect the evolution of an acute seasonal disease. A single El Niño leads to an increase in infections and a decrease in susceptibles during that year. Interestingly, the response of the dynamical disease system to this perturbation leads to a comparable or even larger magnitude impact the year after the El Niño; the infection peak height decreases due to the reduction of the susceptible population during the El Niño the prior year. The effect of a single La Niña event is opposite, with suppressed infections during the La Niña causing susceptibles to build up, which leads to a larger infection peak the year after the La Niña. The interplay between susceptibles and infections causes the effects of the ENSO‐related perturbations to last for multiple additional years, shown by the infected fraction anomaly from the attractor, despite $R_{0}$ returning to its usual seasonal variations.

Figure 3 also shows how the impact of El Niño or La Niña can differ when other ENSO events precede that event. For El Niño events preceded by La Niña, susceptible buildup causes an even higher infection peak during the El Niño than if the El Niño event had existed alone. For La Niña events preceded by El Niño, the opposite occurs. The preceding El Niño drains the susceptibles, leading to an even lower infection peak during the La Niña, and a larger rebound in infections the year after the La Niña. In the cases with three La Niña events preceding an El Niño or three El Niño events preceding La Niña, the effects are similar and sometimes greater, and can be more complex due to the longer perturbation timescale interacting with the infections and susceptibles. Regardless of the sequence of ENSO events, there are substantial multi‐year effects due to lagged effects of susceptible supply on infections.

In addition to changes in peak height, there are shifts in the timing of the infection peaks across these ENSO event scenarios. Across all the experiments, infection peaks shift earlier during El Niño, when $R_{0}$ is high and increasing with seasonality. Infection peaks tend to shift later than usual when $R_{0}$ is low, as during La Niña, because there is a delay in susceptibles growing high enough to enable an outbreak. The infection annual anomalies also show how peak height and annual sum anomalies tend to match in sign and magnitude, but not always. For example, in the 3 El Niño 1 La Niña scenario, there is a lower infection peak height but a higher annual sum of infections due to more off‐peak infections.

**Figure 4:**
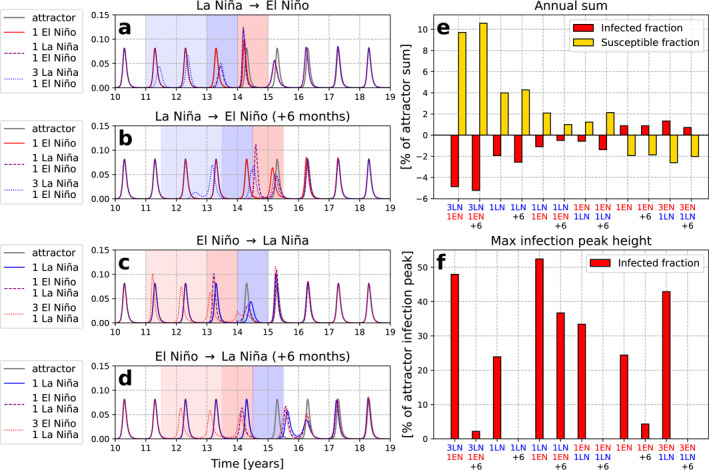
Left: Infected fraction for all seasonal SIRS models with El Niño‐Southern Oscillation (ENSO) perturbations, with attractor in gray. (a) For experiments including El Niño and El Niño preceded by 1 or 3 La Niña events, (b) as (a) but for ENSO perturbations shifted 6 months later, (c) for experiments including La Niña and La Niña preceded by 1 or 3 El Niño events, (d) as (c) but for ENSO perturbations shifted 6 months later. Right: Seasonal ENSO‐SIRS model results for simulated ENSO sequences during model years 11–18 relative to the attractor, as a percentage of that metric for the attractor. Metrics shown are (e) annual sum of infected fraction (red) and susceptible fraction (yellow) and (f) maximum peak infection height. +6 indicates experiments where ENSO perturbations were shifted 6 months later. Experiments are ordered left to right in the general order of negative to positive net $R_{0}$ perturbations, where LN indicates La Niña and EN indicates El Niño, and the numbers indicate the number of events.

The effects of ENSO in the standard timing experiments (Figures 4a and 4c) differ from the 6‐month shifted experiments (Figures 4b and 4d; Figure S1 in Supporting Information S1) due to the difference in timing of the ENSO events relative to the seasonal cycle of $R_{0}$ and susceptible fluctuations. In the standard timing experiments, ENSO‐related $R_{0}$ changes align with increasing seasonal $R_{0}$ and high susceptibles. This primes these models to have large outbreaks during El Niño since all three factors align to enhance infections. In contrast, in the 6‐month shifted experiments, ENSO events begin as $R_{0}$ is seasonally decreasing and susceptibles are low. This primes La Niña to have a large and long‐lasting infection suppression effect. This is demonstrated especially in the 6‐month shifted 3 La Niña 1 El Niño experiment, where one infection peak is extremely low and delayed.

For La Niña in the standard experiments and El Niño in the 6‐month shifted experiments, the ENSO‐related changes in $R_{0}$ are opposed by the seasonal change and susceptible supply, so their effects are subdued. In the standard timing experiments, though La Niña does suppress infections, it is not to the same extent as the 6‐month shifted experiments. In the 6‐month shifted experiments, El Niño is actually associated with smaller outbreaks compared to the attractor. This is because the effect of El Niño on infections does not emerge until the latter half of the event, when $R_{0}$ is seasonally peaking. At that point, susceptibles are at a moderate level which allows an early outbreak, but the outbreaks are small because the susceptibles have not had time to fully build up. The only exception is the 1 La Niña 1 El Niño experiment, which has a large infection peak during El Niño due to the preceding La Niña delaying the peak and causing susceptibles to be high going into the El Niño.

The 6‐month shifted experiments tend to have earlier outbreaks due to El Niño and later outbreaks due to La Niña, similar to the standard timing experiments. The exception is the 3 La Niña 1 El Niño experiment, where the very low infection peak leads to large susceptibles buildup and an early outbreak during the next La Niña.

Figures 4e and 4f summarize how the different ENSO event scenarios impact key measures for infection totals and peak height relative to the attractor, as a percentage of that measure in the attractor. These measures are computed over model years 11–18 because this period encapsulates when the longest ENSO event sequences (i.e., four total events) deviate from the attractor.

Figure 4e shows the annual sum of infections and susceptibles for each ENSO experiment relative to the attractor. For a given variable X, this is computed as:

(1) $$\text{\%}\;\text{change}\;\text{in}\;\text{annual}\;\text{sum}=\left[\frac{sum\left(X_{\text{year}=11‐18}\right)-sum\left(X_{\text{attractor}}\right)}{sum\left(X_{\text{attractor}}\right)}\right]*100$$

Figure 4f compares the highest infection (I) peak height during each ENSO experiment to the attractor and expresses it as a percentage of the attractor peak height, as in the formula below:

(2) $$\text{\%}\;\text{change}\;\text{in}\;\text{highest}\;\text{infection}\;\text{peak}=\left[\frac{\max\left(I_{\text{year}=11‐18}\right)-\max\left(I_{\text{attractor}}\right)}{\max\left(I_{\text{attractor}}\right)}\right]*100$$

If no infection peak height exceeds the attractor peak height for a given scenario, then the value is zero, because the disease returned to the attractor. This metric could be of interest for public health officials because it indicates whether a given ENSO scenario could lead to an unusual surge in infections which may exceed healthcare system capacity.

The annual sum metric (Figure 4e) shows a cleaner picture than the maximum peak height metric, which is perhaps unsurprising because the annual sum is computed over several years rather than a singular value. There is little difference between the standard and 6‐month shifted experiments in the annual sums. El Niño tends to increase the sum of infections and La Niña tends to decrease the sum of infections, which is expected due to our model setup of El Niño increasing $R_{0}$ and La Niña decreasing $R_{0}$.

Interestingly, the magnitude of La Niña's suppression of the annual sum of infections appears to be larger than the increase from El Niño. This asymmetry may be explained by how susceptible supply limits the ability of the El Niño events to increase infections. When $R_{0}$ increases, especially over multiple years, susceptibles drain and lead to smaller outbreaks. During La Niña, although outbreaks occur due to susceptible buildup, the low $R_{0}$ causes infection peaks to be consistently low. This reveals a key nonlinearity about this system; under symmetric changes to $R_{0}$, $R_{0}$ increases are limited in their impact on infections due to low susceptible supply, whereas $R_{0}$ decreases can suppress infections more effectively.

The maximum infection peak height changes are more complex (Figure 4f). Large infection peaks can result from a combination of various factors, such as high background $R_{0}$, high susceptible fraction, and optimal timing of $R_{0}$ increases due to El Niño. Most of the 6‐month shifted experiments (except 1 La Niña 1 El Niño) result in no or little increase in the maximum infection peak height, probably due to the previously discussed opposition between the $R_{0}$ increase from El Niño and the decreasing seasonal $R_{0}$ and low susceptibles.

The rest of the experiments experience maximum infection peak height increases greater than 20% of the attractor peak height. The maximum infection peaks all occur the year after a La Niña event, whether during El Niño or in the neutral state (Figure 4). The La Niña to El Niño transition seems particularly powerful, as the 1 La Niña 1 El Niño scenarios experience the largest increase in infection peak height compared to the other experiments. The La Niña to El Niño transition is accompanied by the largest increase in $R_{0}$. However, what distinguishes the 1 La Niña 1 El Niño scenarios from the 3 La Niña 1 El Niño scenarios is that the former builds up susceptibles and loses them to infections in a shorter amount of time (Figure 3; Figure S1 in Supporting Information S1). In 3 consecutive La Niña events, susceptibles may oscillate more and deplete before the La Niña‐El Niño transition, leading to smaller peaks (e.g., 6‐month shifted 3 La Niña 1 El Niño experiment).

In summary, these results indicate that even for a very stable seasonal disease, ENSO can have multi‐year impacts on infections by setting off multi‐year changes in susceptibles. These results are consistent with previous studies that show how ENSO's influence on infections interplays with nonlinear susceptible dynamics (e.g., Koelle et al., 2005; Pascual et al., 2000, 2008). Additionally, these results demonstrate how the effects of consecutive ENSO events on infections can be more pronounced and complex than for single ENSO events. The impacts of these simulated ENSO perturbations can be asymmetric for El Niño versus La Niña, and are nonlinear in their effects on infection peak amplitude. The timing of the ENSO perturbation to $R_{0}$ relative to the seasonal changes in $R_{0}$ also produces substantially different results.

### 3.2 Biennial SIRS Model

Figures S2–S4 in Supporting Information S1 show the impacts of simulated ENSO perturbations to $R_{0}$ on a biennial disease. The only differences between the biennial and seasonal model are that the biennial model incorporates a longer immunity length (3 years) and double the amount of seasonal $R_{0}$ variability. The ENSO perturbation scenarios tested are the same as for the seasonal disease. $R_{0}$, infected fraction, infected fraction annual anomalies, and susceptible fraction are shown for the standard timing experiments (Figure S2 in Supporting Information S1) and the 6‐month shifted experiments (Figure S3 in Supporting Information S1).

In biennial disease models, stable peaks occur every 2 years. Perturbations to these systems may cause outbreaks to shift earlier or later than expected, and afterward when the system re‐establishes its 2‐year periodicity, the system may stabilize either to the original attractor or a new biennial cycle offset by 1 year. The biennial SIRS ENSO experiments in this study yield both outcomes (Figure S4 in Supporting Information S1), which are summarized in Table 2. All the ENSO sequences that end with La Niña move to the new attractor, whereas only some of the sequences that end with El Niño do. We hypothesize that ending with a La Niña event suppresses infections long enough to shift to the new attractor. Depending on the timing of the perturbation, El Niño appears to shift peaks earlier resulting in a 1‐year offset or keeps the disease timing aligned with the original attractor.

**Table 2:** Summary of Biennial SIRS Model Behavior for Different El Niño‐Southern Oscillation (ENSO) Perturbation Experiments

	La Niña → El Niño	El Niño → La Niña
Returns to original attractor	1 El Niño	None
	1 El Niño +6 months	
	3 La Niña 1 El Niño +6 months	
Moves to offset attractor	1 La Niña 1 El Niño	1 La Niña
		1 La Niña +6 months
	1 La Niña 1 El Niño +6 months	1 El Niño 1 La Niña
		1 El Niño 1 La Niña +6 months
	3 La Niña 1 El Niño	3 El Niño 1 La Niña
		3 El Niño 1 La Niña +6 months

Figure S4 in Supporting Information S1 shows the annual sum and maximum infection peak anomaly from the attractor as a percentage of the attractor for the biennial disease experiments. These are computed the same way as for the seasonal disease (Equations 1 and 2), but the computation period is years 11–22 for the biennial disease.

The infected and susceptible annual sums for the biennial disease (Figure S4e in Supporting Information S1) have some similarities to the seasonal disease (Figure 4e). While the magnitude and sign of the ENSO perturbation‐related changes in annual sum differ for some experiments, the tendency of La Niña events to reduce infections (increase susceptibles) and El Niño events to increase infections (decrease susceptibles) is still present. The more substantial effect of La Niña compared to El Niño is even more pronounced in the biennial disease, as every experiment that includes a La Niña event has a decrease in the sum of infections compared to the attractor. We posit that because the biennial disease has longer times between peaks, the ability of the La Niña perturbations to suppress infections and build up susceptibles is more pronounced than in the seasonal disease.

The maximum infection peak anomalies are positive in all the biennial disease experiments (Figure S4f in Supporting Information S1), including the 6‐month shifted experiments. This is in contrast to the seasonal disease, where the 6‐month shifted experiments did not have as much peak growth (Figure 4f). There is generally more of an increase in the scenarios with La Niña events, with the largest infection peak increase in the 3 La Niña 1 El Niño experiment due to very high susceptibles buildup; however, the same experiment shifted by 6 months does not have as much growth in infections, again indicating the importance of the timing of the ENSO perturbation. Still, it is important that both El Niño and La Niña lead to an increase in infection peak height, either at zero lag or eventually, in the biennial model (as well as most of the seasonal disease experiments).

### 3.3 MERRA‐2/SIRS Model: HCoV‐HKU1

**Figure 5:**
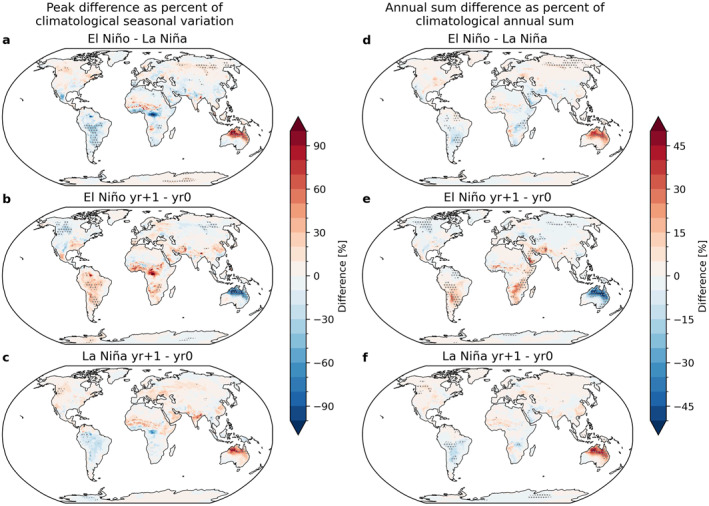
Left: Global maps of differences in infection peak values as a percent of the seasonal variation of infections for an average year (1981–2017). Right: Differences in the annual sum of infections as a percent of the annual sum of infections for an average year (1981–2017). Differences are computed at each individual location for (top row) El Niño minus La Niña, (middle row) El Niño year +1 minus El Niño year 0, and (bottom row) La Niña year +1 minus La Niña year 0. Gray dots indicate significant difference between the means of the two compared data sets at the 95% confidence level. Note that these maps are not weighted by population. Results for El Niño‐Southern Oscillation (ENSO) composite year +2 minus year +1 is shown in Figure S5 of the Supporting Information S1.

In the MERRA‐2/SIRS model, specific humidity data from 1981 to 2017 determines $R_{0}$ in an SIRS model using parameters from HCoV‐HKU1, a human coronavirus (Figure 2). Figure 5 shows how infection peak amplitude and the annual sum of infections are impacted by ENSO phase (El Niño vs. La Niña) as well as the multi‐year impacts of El Niño and La Niña (composite year +1 vs. year 0). Note that these maps are not weighted by population; spatial heterogeneity arises from geographic differences in ENSO teleconnections and specific humidity seasonality influencing infections. Composite year +2 is also compared to year +1 for El Niño and La Niña in Figure S5 of the Supporting Information S1, because most locations in the northern hemisphere tend to have climatological infection peaks earlier in the calendar year, so the first infection peak eligible to be influenced by ENSO events would occur in ENSO composite year +1.

The impact of El Niño versus La Niña on the infection peak height relative to the seasonal variation in infections (Figure 5a) is significant in several regions, including northern Australia, South America, equatorial Africa, and Russia. The difference in infection peak height between El Niño year +1 and year 0 (Figure 5b) is also significant in these locations and northwestern Canada. For La Niña year +1 compared to year 0 (Figure 5c), the effects are more geographically restricted, with northern Australia being particularly impacted, and some parts of South America and western Canada. Differences in infection peak height over 100% of the climatological seasonal cycle of infections are present, especially in Australia.

The impacts of El Niño versus La Niña on the annual sum of infected fraction relative to the climatological annual sum (Figures 5d–5f) generally match the sign of the impacts on infection peak, and more regions emerge as significantly different. The most prominent changes are again in northern Australia. Other significant changes occur in South America, equatorial and east Africa, the Middle East, southeast Asia, western Canada, and Russia.

The larger differences between year +1 and year 0 for El Niño (Figures 5b and 5e) compared to the same for La Niña (Figures 5c and 5f) is likely because El Niño events during this period are often directly followed by La Niña events, whereas La Niña events are often followed by a neutral year, resulting in less contrast. Similar results occur when year +2 is compared to year +1 (Figure S5 in Supporting Information S1), and may be explained for the same reason for locations where there is a lag between ENSO events in year 0 and infection peak timing in year +1 (most of the Northern Hemisphere). In regions where there is not as much of a lag between ENSO and infection peak timing (most of the Southern Hemisphere), there are still differences between the year +2 and year +1 composites, highlighting a significant effect of ENSO events that lasts for 2 years. These 2‐year effects are found in northern and eastern Australia, southeast Africa, and parts of South America and Canada.

**Figure 6:**
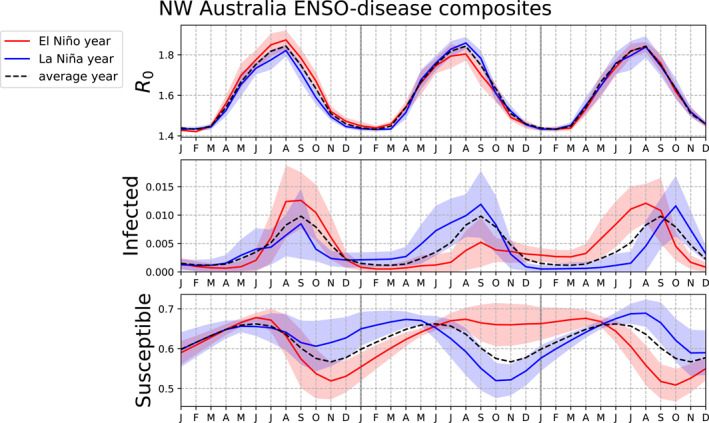
Composites of disease parameters from a location in northwestern Australia (128°E, 18°S) for El Niño (red), La Niña (blue), and the average of all years (black dashed). $R_{0}$ (top row), infected fraction (middle row), and susceptible fraction (bottom row) are shown for year 0 of the El Niño‐Southern Oscillation (ENSO) event, 1 and 2 years after the ENSO event. Shading indicates the 95% confidence intervals for El Niño (pink) and La Niña (light blue).

Since northwestern Australia stands out globally as a location where ENSO impacts infections for all metrics shown and through ENSO composite year +2, this location is examined further. The data shown in Figure 6 are retrieved from a single location in northwestern Australia but are qualitatively similar across the region. Figure 6 shows composites of $R_{0}$, infected fraction, and susceptible fraction during El Niño and La Niña events, and one and 2 years later, compared to the average of all years (climatology). Composites for Neutral years (not shown) are similar to the climatology but not exactly the same, likely because Neutral composite years +1 and +2 often occur during El Niño or La Niña.

In northwestern Australia, El Niño is associated with higher $R_{0}$, leading to higher infections and therefore lower susceptibles, and La Niña is associated with the opposite. As was shown in the global plots, the differences in infection peak amplitude and overall number of infections associated with ENSO events is large in this region compared to the climatology.

The multi‐year impacts of ENSO events on infections are evident, especially the multi‐year role of susceptibles. At this location, impacts of El Niño versus La Niña on infections are stronger in composite year +1 and +2 than during the year of the ENSO event. During composite year +1, the difference in infections between El Niño and La Niña may be explained by both the difference in $R_{0}$ and a difference in susceptibles, though high susceptibles also drive off‐peak infections during the transition from La Niña composite year 0 to +1 and El Niño composite year +1 to +2. During year +2, $R_{0}$ has essentially returned to its climatological values while differences in susceptibles persist, driving a large and earlier infection peak in year +2. These results suggest a large role of susceptible supply for determining infections even 2 years after an ENSO event in this location.

Note that the infections in this location in northwestern Australia are not biennial (see timeseries in Figure S6 of the Supporting Information S1). The biennial peak structure in Figure 6 is associated with the ENSO events, not the baseline disease dynamics.

The same plots are shown for a location in Rondônia, Brazil, which also showed up as significant on the maps in Figure 5 and where La Niña and El Niño are associated with the opposite sign of changes in $R_{0}$ (Figures S7 and S8 in Supporting Information S1). Though this location is less dramatically affected by ENSO and has shallower disease peaks than northwestern Australia, the multi‐year role of susceptibles is also evident in this location, even when $R_{0}$ has returned to its climatological values during composite year +2. Much of the El Niño‐La Niña infections difference here occurs during the off‐peak season, affecting the infection sum rather than the peak height, and demonstrating how ENSO's impacts may differ in locations with different underlying disease dynamics.

## 4 Discussion and Conclusions

The responses of seasonal and biennial SIRS models to idealized ENSO perturbations, along with the ENSO composites for HCoV‐HKU1 in the MERRA‐2/SIRS model, reveal the potential impacts of ENSO on climate‐driven infectious diseases with immunity length greater than 1 year. Both the idealized and weather data‐based modeling approaches suggest that ENSO can exert multi‐year effects on disease dynamics due to lagged changes in susceptibles, which is consistent with previous studies (e.g., Koelle et al., 2005; Pascual et al., 2008).

A major finding across all the models is that both El Niño and La Niña can be associated with increases in infection peak height, either during the event or at a lag due to susceptibles buildup. This implies that interventions to offset the growth in susceptibles (e.g., immunization) could significantly help avoid major spikes in infections. We also found several instances of outbreaks shifting earlier or later, and understanding these dynamics better may help inform the timing of interventions. Additionally, our results repeatedly found larger effects of ENSO on infections a year or more after the ENSO events, implying that understanding ENSO‐disease relationships requires accounting for susceptible dynamics.

Our findings also highlight the importance of considering how consecutive ENSO events may impact susceptibles and infections rather than focusing solely on a single ENSO event. A potential application of this finding could be when ENSO‐sensitive disease outbreaks vary in size for ENSO events of similar magnitude. Different population immunity levels likely drive these differences (e.g., Pascual et al., 2008), therefore for diseases with long immunity timescales, the effects of preceding consecutive ENSO events on susceptibles may be worth considering. This complexity suggests that disease data and analysis must encompass various sequences of ENSO events to fully grasp how ENSO may affect disease dynamics, motivating multi‐decadal data collection.

The impacts of ENSO on infections were also sensitive to the underlying disease dynamics and timing of the ENSO‐associated change in $R_{0}$ relative to seasonal changes in $R_{0}$. This could explain some ENSO sensitivity differences across different locations in the MERRA‐2/SIRS model. Future work could group locations with similar disease dynamics (e.g., seasonal, biennial, shallow peaks) or based on the timing of their climatological infection peaks relative to ENSO‐induced changes in $R_{0}$, and investigate the multi‐year impacts of ENSO on each group in further detail. Our results also motivate better simulation of the seasonal phase‐locking of ENSO and its teleconnections in climate models and projections.

An intriguing result from the idealized models is the asymmetry between El Niño and La Niña events, with La Niña events having a more pronounced effect in reducing disease burden compared to El Niño events increasing it. This warrants further exploration, although caution is advised when using real‐world data due to potential confounding factors, such as differing sequences of ENSO events.

It is crucial to recognize the limitations of modeling when attempting to bridge the gap between simulated outcomes and real‐world scenarios. Although nearly 40 years of data were used in the MERRA‐2/SIRS model, considerable variability was observed between ENSO events, likely because they are diverse in their strength, timing, character, and teleconnections. Future work could investigate how extreme and moderate ENSO events, east and central Pacific El Niño events, and variations in ENSO teleconnections might influence disease dynamics differently, possibly leveraging global climate models to gain a larger sample, if the teleconnections are well‐represented.

Factors such as human behavior, healthcare interventions, and weather patterns unrelated to ENSO can also mask the effects of ENSO on disease dynamics. Also, the details of the relationship between specific humidity and $R_{0}$ upon which the MERRA‐2/SIRS model relies is based on United States HCoV‐HKU1 data and may be modulated by local context. Future work aimed at more realistic simulation of disease spread should account for these potential geographic differences in disease dynamics.

These results present several intriguing avenues for future research. The choice to model a respiratory disease in the MERRA‐2/SIRS model was motivated by the strong seasonality and humidity dependence commonly associated with such diseases (Baker et al., 2019; Lowen & Steel, 2014; Lowen et al., 2007; Shaman & Kohn, 2009; Shaman et al., 2010). Although airborne diseases have not been as much of a focus of ENSO‐disease research compared to vector and water‐borne diseases, this study's findings suggest the potential importance of multi‐year effects of ENSO and different ENSO event sequences for airborne diseases. This motivates more nuanced temporal investigation into the impact of ENSO on airborne diseases and may be relevant for other types of environmentally influenced diseases with long immunity timescales. Additionally, this study leveraged the SIRS framework. Given the assumed short duration of immunity, this model is a reasonable approximation of recurrent epidemic dynamics. However, the impact of ENSO on the full family of SIR models (with births/deaths) should be investigated in future research (e.g., Keeling & Rohani, 2008).

Changes in ENSO associated with anthropogenic climate change or multi‐decadal variability introduce uncertainty into ENSO‐disease relationships. Climate models could offer insight into these potential shifts, although careful consideration is warranted regarding the realism of ENSO changes in these models. Additionally, population distribution and demographic changes, which were ignored in this study, play a crucial role in understanding vulnerability to disease. Overlaying population data onto ENSO‐disease forecasting models could help identify regions most vulnerable to ENSO‐related disease outbreaks.

This study's findings also have several implications for ENSO‐disease forecasting. While documenting infections, vaccination status, and estimating susceptible populations are challenging tasks, understanding ENSO teleconnections to different regions offers a more straightforward opportunity based in climate physics rather than human behavior. To understand the interaction between ENSO and disease, it is imperative to consider the sequences of ENSO events, not only individual events. If these climate and disease interactions become well‐understood, interventions could be planned several months in advance, leveraging the multi‐month lead time of ENSO forecasting and the multi‐season changes in population immunity.

## Conflict of Interest

The authors declare no conflicts of interest relevant to this study.

## Supporting information

Supporting Information S1

## Data Availability

The code for running the models and analysis in this work and the idealized SIRS model output are published on Github and licensed under MIT (Chung, 2024a). The MERRA‐2/SIRS model specific humidity input data and disease variable output data is available for download on Zenodo (Chung, 2024b) under a Creative Commons Attribution 4.0 International license. The raw MERRA‐2 data is available for download from NASA (Global Modeling And Assimilation Office (GMAO), 2015).
