# Supplementary material for: Intersecting Memories of Immunity and Climate: Potential Multiyear Impacts of the El Niño–Southern Oscillation on Infectious Disease Spread
Source: Geohealth. 2025 Feb 11;9(2):e2024GH001193. doi: 10.1029/2024GH001193 (PMC11811887; doi:10.1029/2024GH001193)
Supplement: Supplementary file 1 — Supporting Information S1 [file GH2-9-e2024GH001193-s001.pdf]

GeoHealth

Supporting information for

Intersecting memories of immunity and climate: Potential multiyear impacts of the El Niño—Southern Oscillation on infectious disease spread

Maya V. Chung[1,2], Gabriel A. Vecchi[1,2,3], Wenchang Yang[3], Bryan Grenfell[2,4,5], C. Jessica Metcalf[2,4,5]

[1]*Program in Atmospheric and Oceanic Sciences, Princeton University*

[2]*High Meadows Environmental Institute, Princeton University*

[3]*Department of Geosciences, Princeton University*

[4]*Department of Ecology and Evolutionary Biology, Princeton University*

[5]*Princeton School of Public and International Affairs*

Contents of this file

Figures S1 to S8

Introduction

Figures S1, S2, and S3 are the equivalent of Figure 3 in the main text but for the seasonal SIRS model shifted 6 months later, and the biennial model and biennial shifted 6 months later.

Figure S4 is the equivalent of Figure 4 but for the biennial SIRS models.

Figure S5 is similar to Figure 5 but compares ENSO composite years +1 and +2 instead of El Niño vs. La Niña and ENSO composite years 0 and +1.

Figure S6 shows the timeseries of disease parameters for the location in northwestern Australia shown in Figure 6, to illustrate the timeseries of infections and their overlap with ENSO events.

Figures S7 and S8 are the equivalents of Figure 6 and Figure S6 but for a location in western Brazil where the influence of ENSO on disease spread is the reverse sign from the northwestern Australia location.

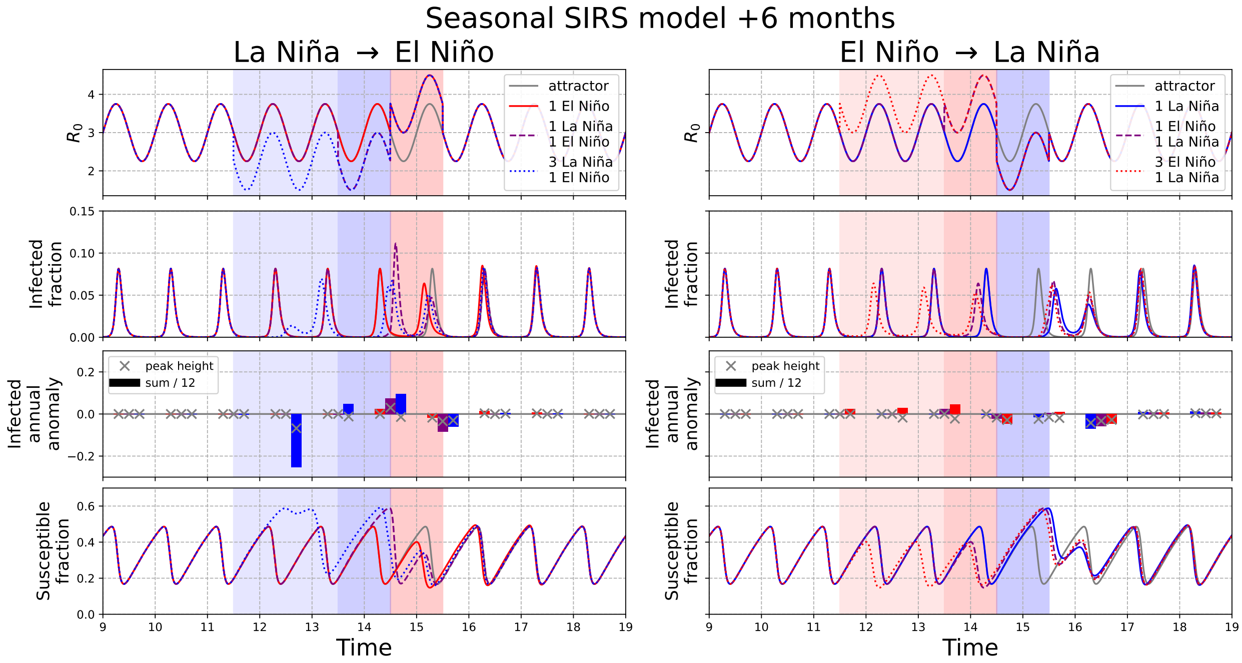

Figure S1: As Figure 3, for ENSO perturbations timed 6 months later.
SIRS model response to ENSO perturbations for El Niño and preceding 1 or 3 La Niña events (left) and La Niña and preceding 1 or 3 El Niño events (right). All the models have seasonally-varying R_0, with no perturbations to the attractor (gray), and with simulated El Niño (pink shading) and La Niña events (blue shading) as a one-year R_0 increase or decrease from the seasonally-varying baseline, respectively. Darker shading indicates the overlap in timing between the 1 and 3 preceding-event scenarios. Quantities shown over time are R_0, infected fraction, infected fraction annual anomaly from the attractor as the difference in peak height ($\times$) and annual sum divided by 12 (bars), and susceptible fraction. Colors for the infected annual anomalies correspond to the ENSO event scenarios with line plots of the same color. Left: One El Niño event (red solid line), La Niña followed by El Niño (purple dashed line), and three La Niña events followed by El Niño (blue dotted line). Right: One La Niña event (blue line), El Niño followed by La Niña (purple dashed line), and three El Niño events followed by La Niña (red dotted line).

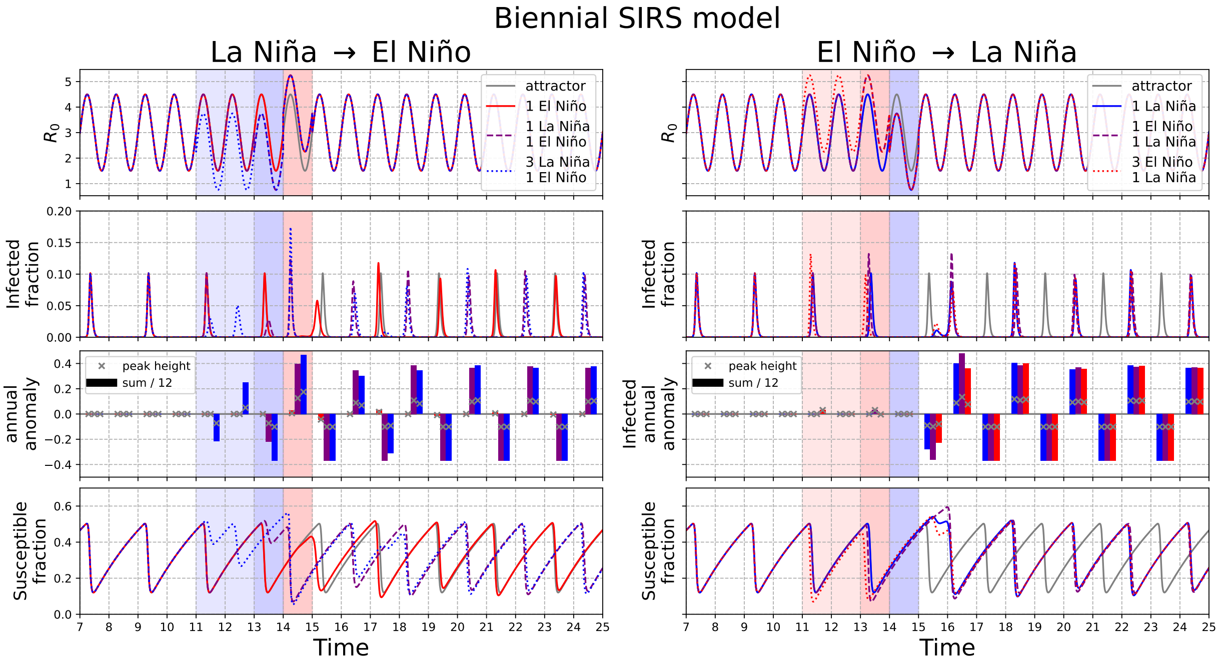

Figure S2: As Figure 3, for the biennial model.
SIRS model response to ENSO perturbations for El Niño and preceding 1 or 3 La Niña events (left) and La Niña and preceding 1 or 3 El Niño events (right). All the models have seasonally-varying R_0, with no perturbations to the attractor (gray), and with simulated El Niño (pink shading) and La Niña events (blue shading) as a one-year R_0 increase or decrease from the seasonally-varying baseline, respectively. Darker shading indicates the overlap in timing between the 1 and 3 preceding-event scenarios. Quantities shown over time are R_0, infected fraction, infected fraction annual anomaly from the attractor as the difference in peak height ($\times$) and annual sum divided by 12 (bars), and susceptible fraction. Colors for the infected annual anomalies correspond to the ENSO event scenarios with line plots of the same color. Left: One El Niño event (red solid line), La Niña followed by El Niño (purple dashed line), and three La Niña events followed by El Niño (blue dotted line). Right: One La Niña event (blue line), El Niño followed by La Niña (purple dashed line), and three El Niño events followed by La Niña (red dotted line).

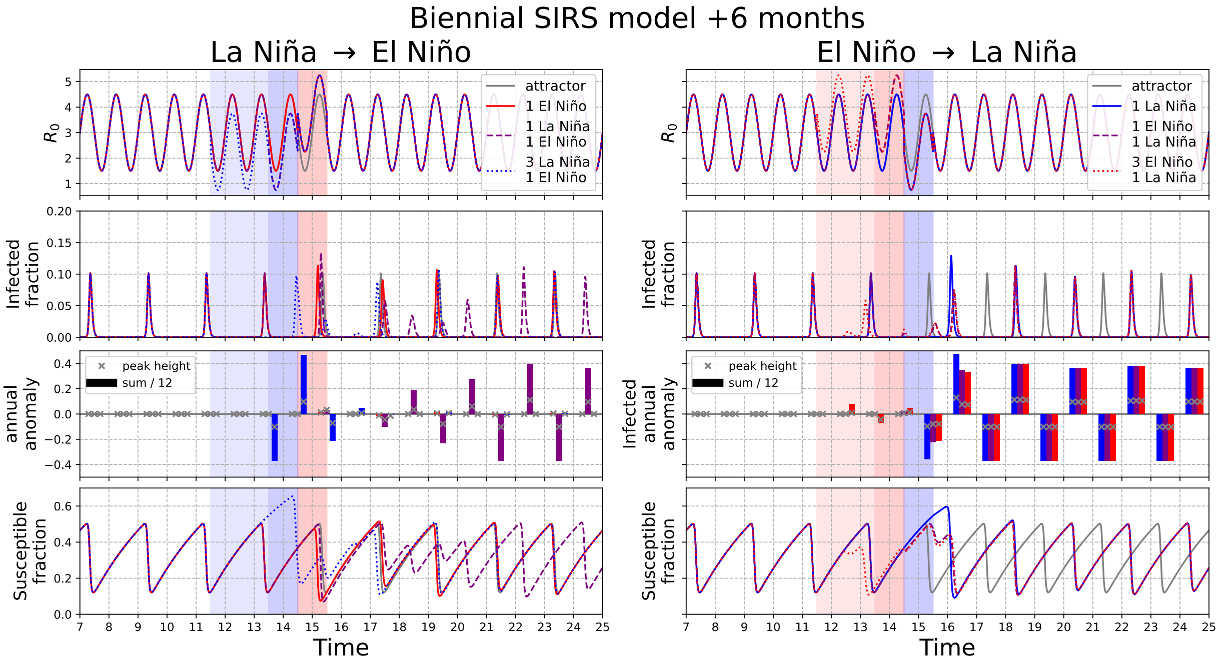

Figure S3: As Figure 3, for the biennial model with ENSO perturbations timed 6 months later. SIRS model response to ENSO perturbations for El Niño and preceding 1 or 3 La Niña events (left) and La Niña and preceding 1 or 3 El Niño events (right). All the models have seasonally-varying R_0, with no perturbations to the attractor (gray), and with simulated El Niño (pink shading) and La Niña events (blue shading) as a one-year R_0 increase or decrease from the seasonally-varying baseline, respectively. Darker shading indicates the overlap in timing between the 1 and 3 preceding-event scenarios. Quantities shown over time are R_0, infected fraction, infected fraction annual anomaly from the attractor as the difference in peak height ($\times$) and annual sum divided by 12 (bars), and susceptible fraction. Colors for the infected annual anomalies correspond to the ENSO event scenarios with line plots of the same color. Left: One El Niño event (red solid line), La Niña followed by El Niño (purple dashed line), and three La Niña events followed by El Niño (blue dotted line). Right: One La Niña event (blue line), El Niño followed by La Niña (purple dashed line), and three El Niño events followed by La Niña (red dotted line).

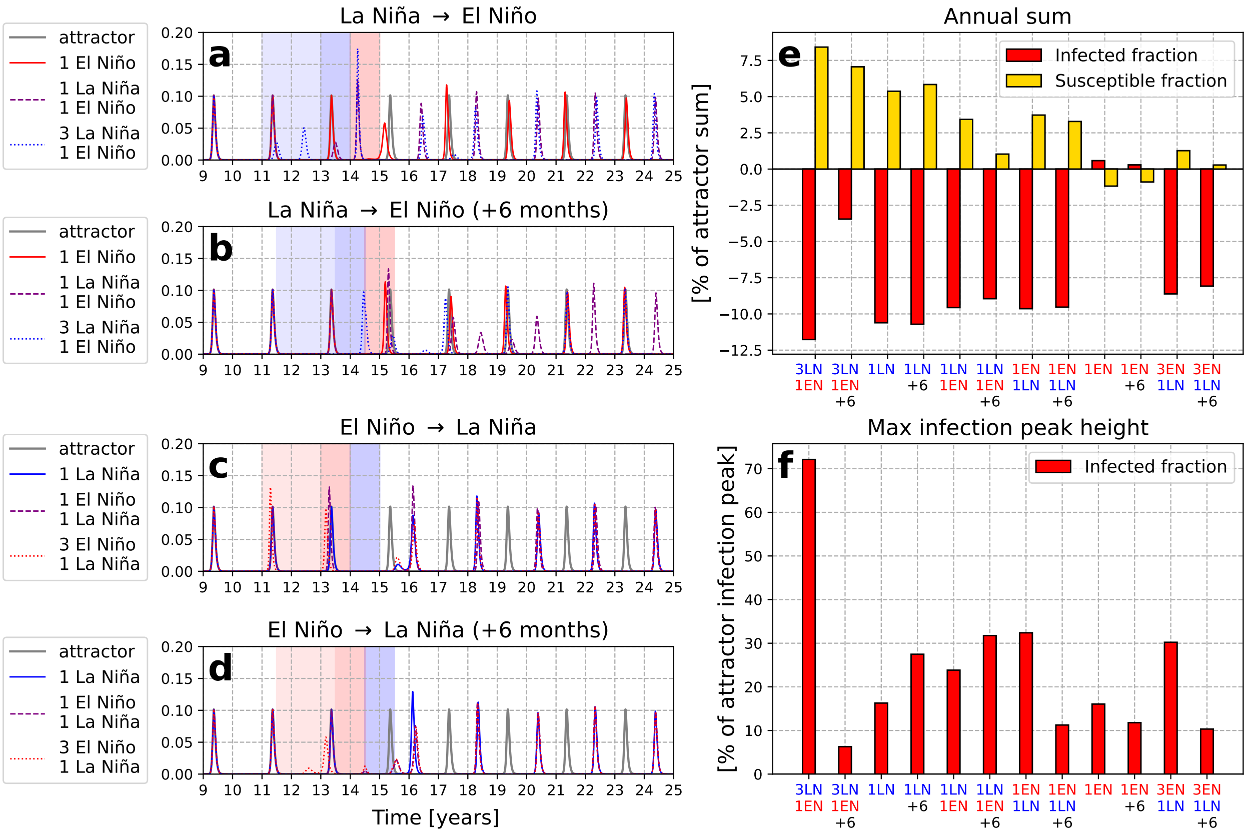

Figure S4: As Figure 4, for the biennial SIRS models.
Left: Infected fraction for all biennial SIRS models with ENSO perturbations, with attractor in grey. (a) For experiments including El Niño and El Niño preceded by 1 and 3 La Niña events, (b) as (a) but for ENSO perturbations shifted 6 months later, (c) for experiments including La Niña and La Niña preceded by 1 or 3 El Niño events, (d) as (c) but for ENSO perturbations shifted 6 months later. Right: Seasonal ENSO-SIRS model results for simulated ENSO sequences during model years 11-22 relative to the attractor, as a percentage of that metric for the attractor. Metrics shown are (e) annual sum of infected fraction (red) and susceptible fraction (yellow) and (f) maximum peak infection height. +6 indicates experiments where ENSO perturbations were shifted 6 months later. Experiments are ordered left to right in the general order of negative to positive net R_0 perturbations, where LN indicates La Niña and EN indicates El Niño, and the numbers indicate the number of events.

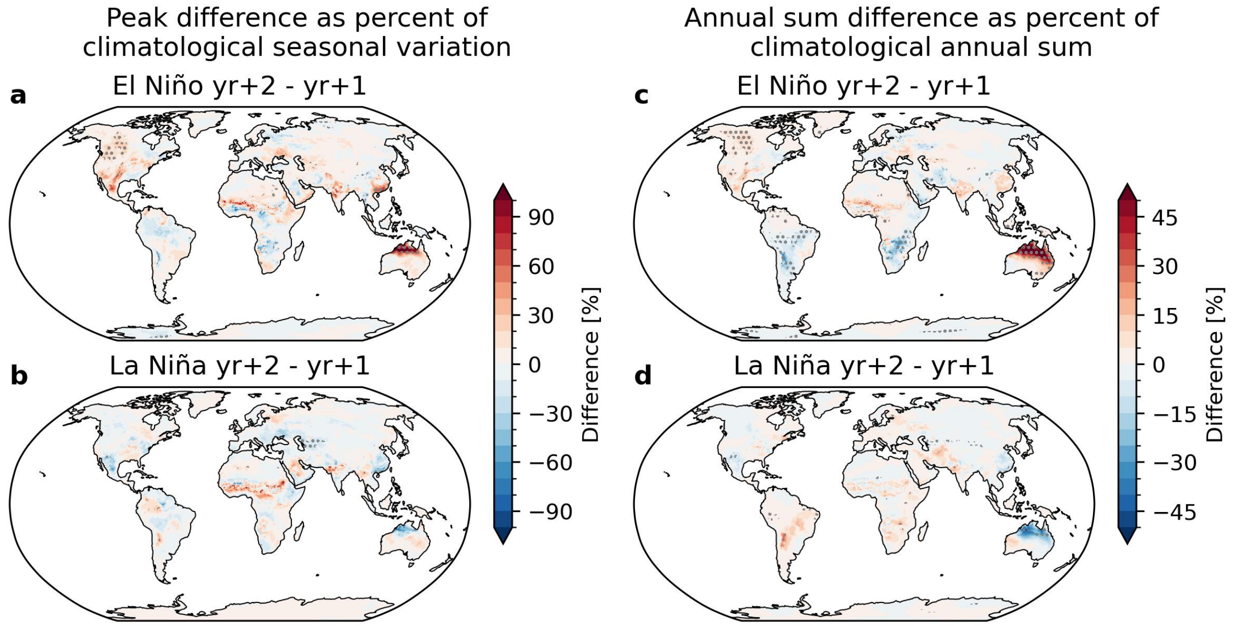

Figure S5: As Figure 5, for El Niño and La Niña composite year +2 minus year +1.
Left: Global maps of differences in infection peak values as a percent of the seasonal variation of infections for an average year (1981-2017). Right: Differences in the annual sum of infections as a percent of the annual sum of infections for an average year (1981-2017). Differences are computed at each individual location for (top row) El Niño year +1 minus El Niño year 0, and (bottom row) La Niña year +1 minus La Niña year 0. Gray dots indicate significant difference between the means of the two compared datasets at the 95% confidence level. Note that these maps are not weighted by population.

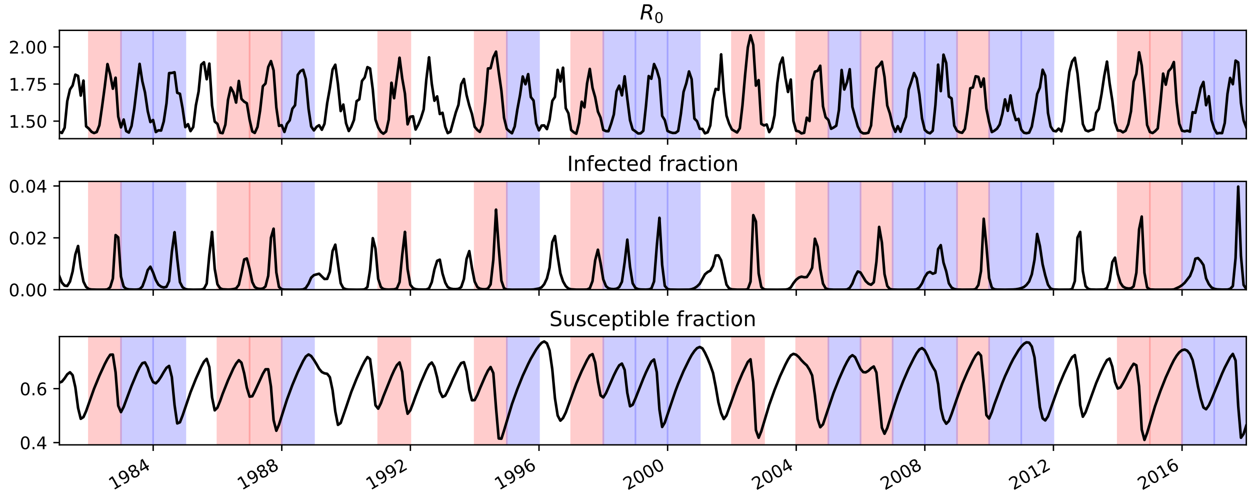

Figure S6: R_0, infected fraction, and susceptible fraction for the location in northwestern Australia shown in Figure 6 (128°E, 18°S). Red and blue shading indicate El Niño and La Niña years according to the ONI index, while white is neutral years.

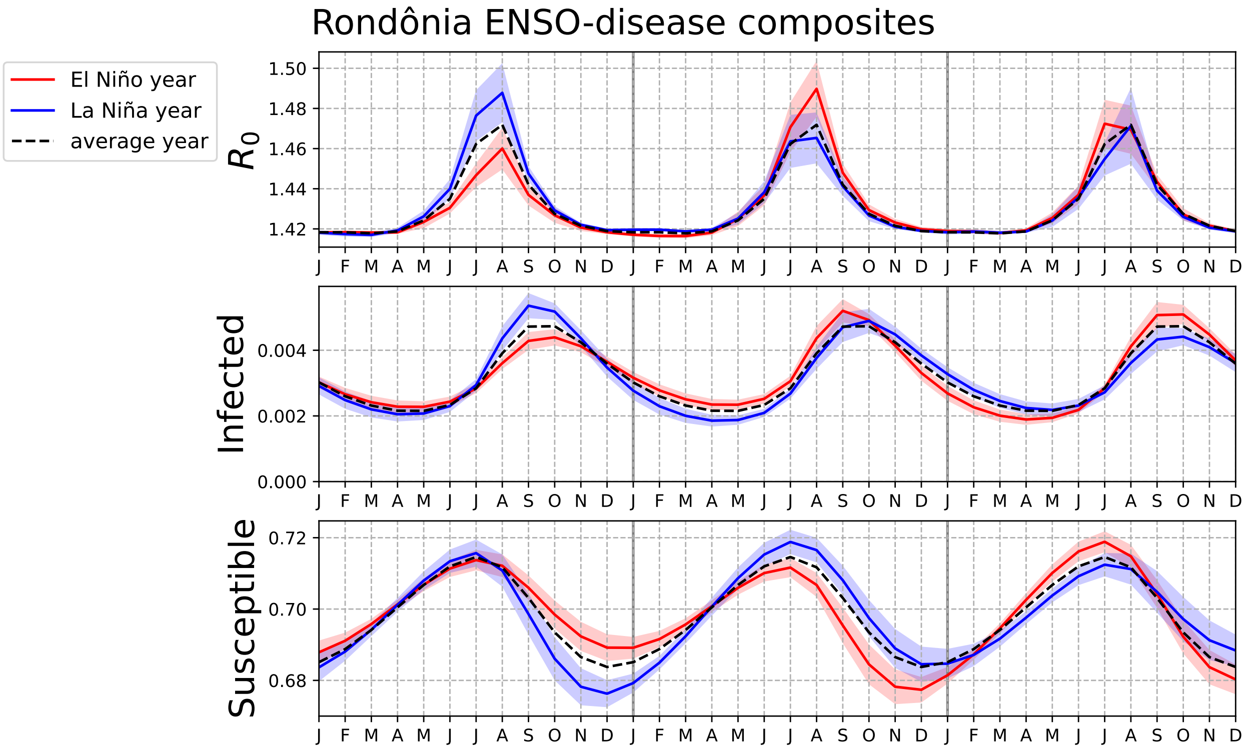

Figure S7: As Figure 6 for a location in Rondônia, a state in western Brazil (63°W, 11°S). Composites of disease parameters for El Niño (red), La Niña (blue), and the average of all years (black dashed). R_0 (top row), infected fraction (middle row), and susceptible fraction (bottom row) are shown for year 0 of the ENSO event, 1 and 2 years after the ENSO event. Shading indicates the 95% confidence intervals for El Niño (pink) and La Niña (light blue).

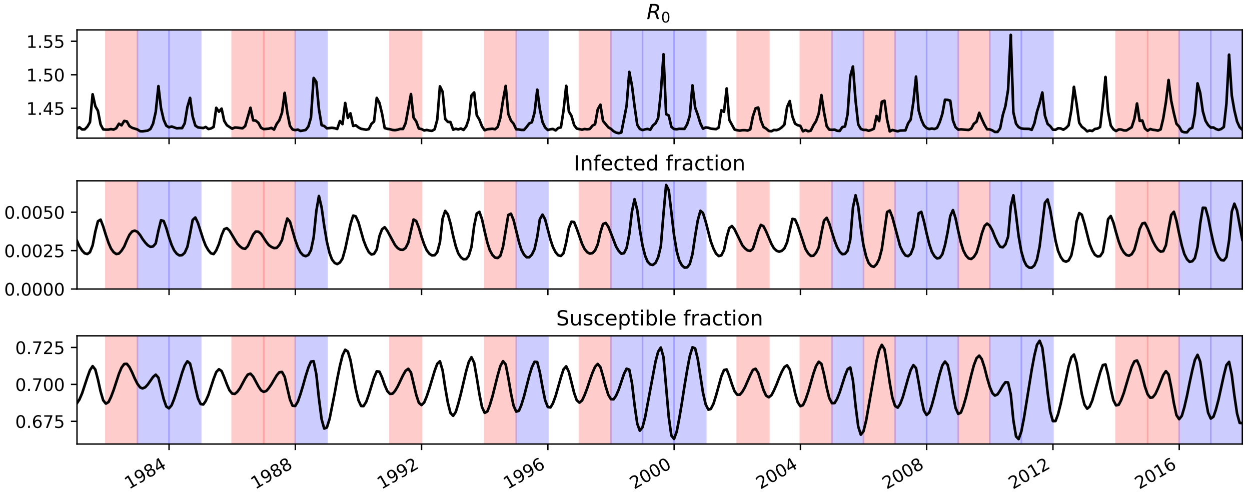

Figure S8: As Figure S6 for a location in Rondônia, Brazil (63°W, 11°S).
